# 2,2′-(Disulfanedi­yl)di­benzoic acid *N*,*N*-di­methyl­formamide monosolvate: crystal structure, Hirshfeld surface analysis and computational study

**DOI:** 10.1107/S2056989020008257

**Published:** 2020-06-26

**Authors:** Sang Loon Tan, Edward R. T. Tiekink

**Affiliations:** a Research Centre for Crystalline Materials, School of Science and Technology, Sunway University, 47500 Bandar Sunway, Selangor Darul Ehsan, Malaysia

**Keywords:** crystal structure, 2,2′-di­thiodi­benzoic acid, di­methyl­formamide, hydrogen bonding, Hirshfeld surface analysis, computational chemistry

## Abstract

The title 1:1 solvate, C_14_H_10_O_4_S_2_·C_3_H_7_NO, features a twisted mol­ecule of 2,2′-di­thiodi­benzoic acid (DTBA), with the central C—S—S—C torsion angle being −88.57 (6)°, and a mol­ecule of di­methyl­formamide (DMF). The carb­oxy­lic acid groups are, respectively, close to co-planar and twisted with respect to the benzene rings to which they are connected as seen in the CO_2_/C_6_ torsion angles of 1.03 (19) and 7.4 (2)°. Intra­molecular, hypervalent S←O inter­actions are noted [S⋯O = 2.6140 (9) and 2.6827 (9) Å]. In the crystal, four-mol­ecule aggregates are formed *via* DTBA-O—H⋯O(DMF) and DTBA-O—H⋯O(DTBA) hydrogen bonding, the latter *via* an eight-membered {⋯OHCO}_2_ homosynthon. These are linked into supra­molecular layers parallel to (011) *via* benzene-C—H⋯O(DTBA) and DTBA-C=O⋯π(benzene) inter­actions, with the connections between these, giving rise to a three-dimensional architecture, being of the type benzene-C—H⋯π(benzene). An analysis of the calculated Hirshfeld surfaces indicates, in addition to the aforementioned inter­molecular contacts, the presence of stabilizing inter­actions between a benzene ring and a quasi-*π*-system defined by O—H⋯O hydrogen bonds between a DTBA dimer, *i.e*. the eight-membered {⋯OCOH}_2_ ring system, and between a benzene ring and a quasi-π(OCOH⋯OCH) system arising from the DTBA-O—H⋯O(DMF) hydrogen bond. The inter-centroid separations are 3.65 and 3.49 Å, respectively.

## Abstract 2

In the title 1:1 solvate, 2,2′-di­thiodi­benzoic acid (DTBA):di­methyl­formamide (DMF), the DTBA mol­ecule is twisted [C—S—S—C = −88.57 (6)°]. Four-mol­ecule aggregates are formed in the crystal *via* DTBA-O—H⋯O(DMF) and DTBA-O—H⋯O(DTBA) hydrogen bonding. These are connected in three-dimensions by benzene-C—H⋯O(DTBA), DTBA-C=O⋯π(benzene) and benzene-C—H⋯π(benzene) inter­actions.

## Chemical context   

Co-crystal formation with 2-mercapto­benzoic acid (2-MBA) is fraught as during crystallization, this is usually oxidized to 2,2′-di­thiodi­benzoic acid (DTBA) (Broker & Tiekink, 2007 ▸; Broker *et al.*, 2008 ▸). Indeed, the only co-crystal of 2-MBA is that with DTBA (Rowland *et al.*, 2011 ▸). With this chemistry in mind, in recent times it has proved possible to isolate co-crystals of DTBA with other carb­oxy­lic acids, such as with a variety of benzoic acid (BA) derivatives, but not always with control over the stoichiometry. Thus, under very much the same conditions, the 1:1 DTBA:BA co-crystal has been characterized (Tan & Tiekink, 2019*a* ▸) along with 2:1 DTBA co-crystals with 3-chloro­benzoic acid (3-ClBA) (Tan & Tiekink, 2019*b* ▸) and the bromo (3-BrBA) analogue (Tan & Tiekink, 2019*c* ▸). The common supra­molecular feature of these crystals is the formation of eight-membered {⋯HOCO}_2_ synthons, occurring between like and/or unlike carb­oxy­lic acids. In a recent study, it was found the anti­cipated {⋯HOCO}_2_ synthon was not always formed but was usurped by a DTBA-O—H⋯O(DMF) hydrogen bond for one of the carb­oxy­lic acids, *i.e*. in the 1:1:1 co-crystal solvate DTBA:2-ClBA:DMF (Tan & Tiekink, 2019*d* ▸); DMF is di­methyl­formamide. It turns out the same situation is noted in the structure of the DTBA:2DMF solvate (Cai *et al.*, 2006 ▸; Ma *et al.*, 2013 ▸; Baruah, 2016 ▸) where the DMF mol­ecule effectively blocks off the capacity for {⋯HOCO}_2_ synthon formation by DTBA. In our hands, recrystallization of 2-MBA from a benzene/DMF (1 ml/7 ml *v*/*v*) solution also gave the DTBA:2DMF solvate (Tan & Tiekink, 2020 ▸). However, an analogous experiment from a benzene/DMF (5 ml/1 ml *v*/*v*) solution yielded the mono-solvate, *i.e*. the title compound DTBA:DMF, (I). The crystal and mol­ecular structures of (I) are described herein along with an analysis of the calculated Hirshfeld surfaces and a computational chemistry study.
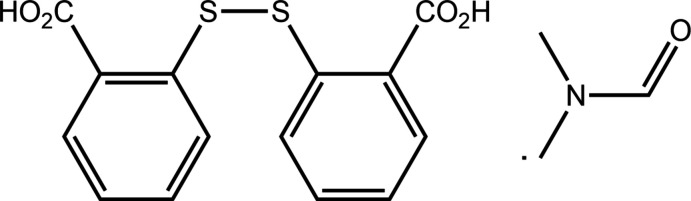


## Structural commentary   

**Figure 1:**
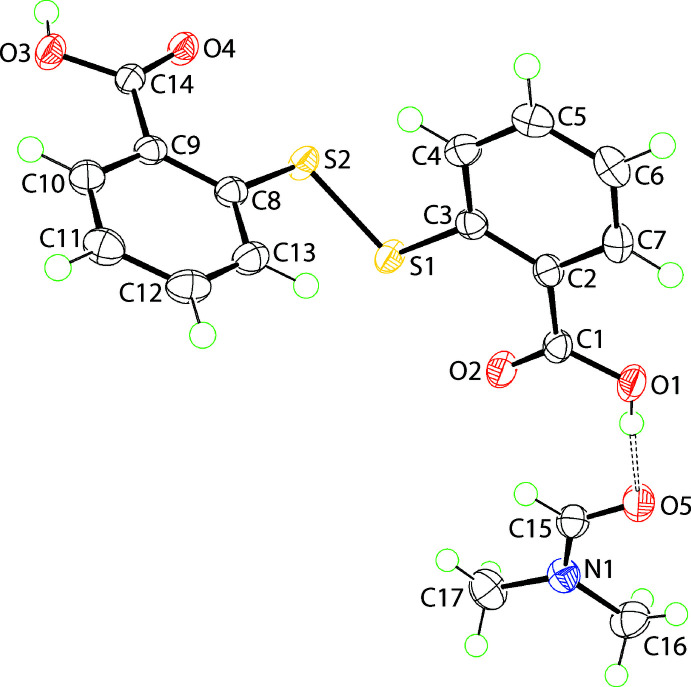
The mol­ecular structures of the constituents of (I) showing the atom-labelling scheme and displacement ellipsoids at the 70% probability level. The dashed line indicates a hydrogen bond.

The asymmetric unit of (I) comprises a mol­ecule of di­thiodi­benzoic acid (DTBA) and di­methyl­formaide (DMF), each in a general position, Fig. 1 ▸. The crystals were obtained from the recrystallization of 2-mercapto­benzoic acid from a benzene/DMF (5 ml/1 ml *v*/*v*) solution indicating the acid oxidized to DTBA during crystallization. The observed disparity in the C—O bond lengths in the carb­oxy­lic acid residues [C1—O1,O2 = 1.3177 (15) & 1.2216 (15) Å and C14—O3,O4 = 1.3184 (14) & 1.2295 (14) Å] confirms the location of the acidic H atoms on the O1 and O3 atoms, respectively. A characteristic twisted conformation is evidenced in the C3—S1—S2—C8 torsion angle of −88.57 (6)°. The dihedral angle between the benzene rings is 87.71 (3)°, consistent with an orthogonal disposition. The C1-carb­oxy­lic acid group is almost co-planar with the (C2–C7) benzene ring to which it is connected with the dihedral angle between the least-squares planes being 1.03 (19)°. By contrast, a small twist is noted for the C14-carb­oxy­lic acid residue where the comparable dihedral angle is 7.4 (2)°. Intra­molecular hypervalent S←O inter­actions (Nakanishi *et al.*, 2007 ▸) are indicated as the carbonyl-O2 and O4 atoms are orientated towards the di­sulfide-S1 and S2 atoms, respectively, with the S1⋯O2 and S2⋯O4 separations being 2.6140 (9) and 2.6827 (9) Å, respectively.

## Supra­molecular features   

**Figure 2:**
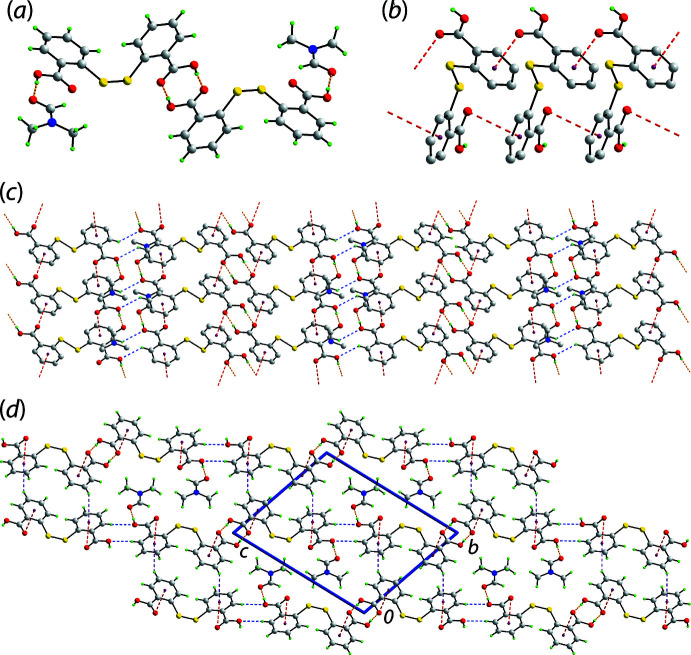
Mol­ecular packing in the crystal of (I): (*a*) the four-mol­ecule aggregate sustained by DTBA-O—H⋯O(DMF) and DTBA-O—H⋯O(DTBA) hydrogen bonding shown as orange dashed lines, (*b*) the supra­molecular chain sustained by carbonyl-O⋯π(benzene) inter­actions shown as red dashed lines, (*c*) the supra­molecular layer with benzene-C—H⋯O(DTBA) inter­actions shown as blue dashed lines and (*d*) a view of the unit-cell contents down the *a* axis with benzene-C—H⋯π(benzene) inter­actions shown as purple dashed lines. In (*b*) and (*c*) the non-participating H atoms have been omitted to aid clarity.

The key feature of the supra­molecular aggregation in the crystal of (I) is the formation of hydrogen bonds between the DTBA-hydroxyl-O1 and the DMF-O5 atoms, as indicated in Fig. 1 ▸ and detailed in Table 1 ▸, along with hydrogen bonds between centrosymmetrically related C14-carb­oxy­lic acid groups associating *via* an eight-membered {⋯OHCO}_2_ homosynthon. The result is the four-mol­ecule aggregate shown in Fig. 2 ▸(*a*). For the DTBA⋯DMF inter­action, further stabilization is realized through a DMF-C15—H⋯O2(carbon­yl) contact, Table 1 ▸, to close a seven-membered {⋯HOCO⋯HCO} heterosynthon. This cooperativity accounts for the near co-planar relationship between the C1-carb­oxy­lic acid group and the non-H atoms of the DMF mol­ecule (r.m.s. deviation = 0.0125 Å) as seen in the dihedral angle of 10.21 (19)° between the two residues. The four-mol­ecule aggregates are linked into supra­molecular chains *via* benzene-C7—H⋯O(hydrox­yl) inter­actions occurring between centrosymmetrically related mol­ecules. The chains are connected by parallel C=O⋯π(benzene) inter­actions as detailed in Fig. 2 ▸(*b*) and Table 1 ▸. The resulting supra­molecular layer is parallel to (011), Fig. 2 ▸(*c*), with connections between them leading to a three-dimensional architecture being benzene-C11—H⋯π(benzene), Fig. 2 ▸(*d*).

**Figure 3:**
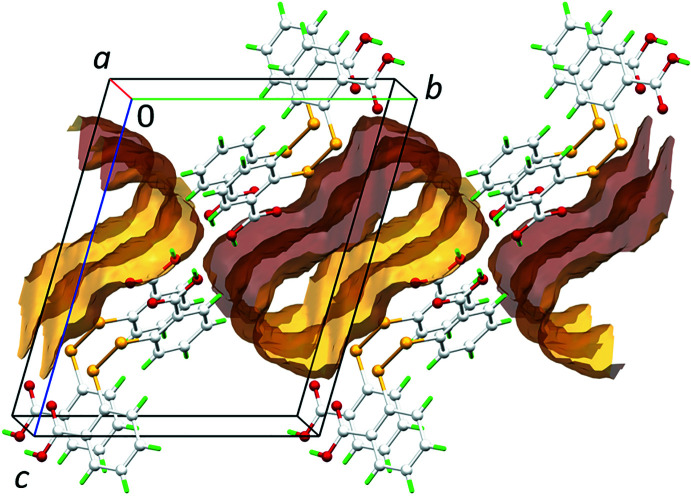
A perspective view of the solvent-accessible voids in the crystal of (I), calculated after removal of the DMF solvent mol­ecules within 2 × 2 × 1 unit-cells.

Crystal (I) was also subjected to the calculation of solvent-accessible void space through *Mercury* (Macrae *et al.*, 2020 ▸) with a probing radius of 1.2 Å within an approximate grid spacing of 0.3 Å. It was found that the DMF solvent mol­ecules occupy about 25.4% or equivalent to 220.8 Å^3^ of the unit-cell volume, whereas the remaining 74.6% or equivalent to 649.2 Å^3^ is occupied by DTBA mol­ecules, as highlighted in Fig. 3 ▸.

## Hirshfeld surface analysis   

**Figure 4:**
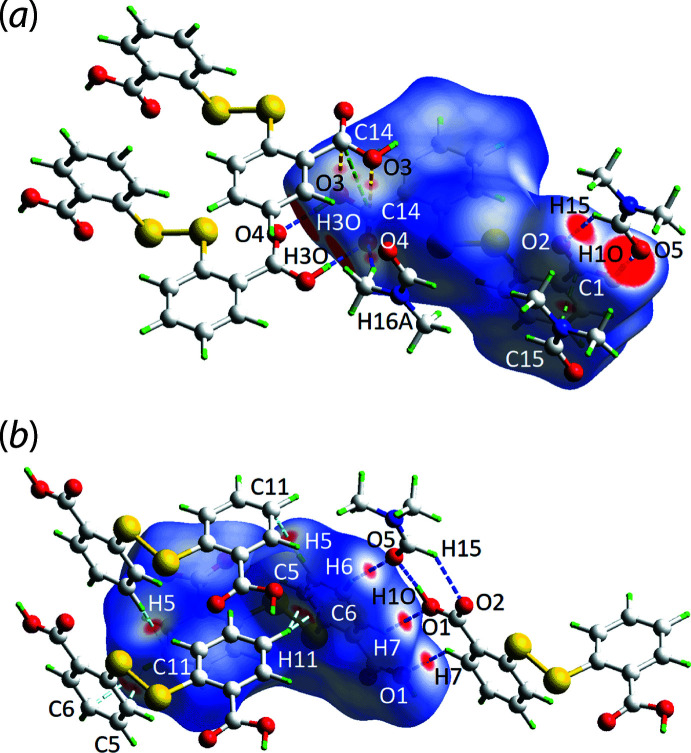
Two views of the *d*_norm_ map for the DTBA mol­ecule, showing the relevant short contacts indicated by the red spots on the Hirshfeld surface with varying intensities within the range of −0.0140 to 1.0154 arbitrary units for (*a*) H3*O*⋯O4, H1*O*⋯O5, H15⋯O2, C15⋯C1, C14⋯O3, C14⋯C14 and H16*A*⋯O4 and (*b*) H6⋯O5, H7⋯O1, H5⋯C11, H11⋯C5 and H11⋯C6. All H⋯O/O⋯H inter­actions are indicated in blue, H⋯C/C⋯H in light-blue, C⋯O/O⋯C in yellow and C⋯C in green. The close contacts present in the DMF mol­ecule mirror that of the DTBA and hence the relevant *d*_norm_ maps are not shown.

To better comprehend the supra­molecular features of (I), it was subjected to Hirshfeld surface analysis through *Crystal Explorer 17* (Turner *et al.*, 2017 ▸) using the established methods (Tan *et al.*, 2019 ▸). Several close contacts with distances shorter than the sum of van der Waals radii (Spackman & Jayatilaka, 2009 ▸) are manifested by red spots of varying intensities on the Hirshfeld surface calculated over *d*_norm_ in Fig. 4 ▸. Specifically, the most intense red spots are noted for hy­droxy-O1—H1*O*⋯O5(carbon­yl) and hy­droxy-O3—H3*O*⋯O4(carbon­yl) hydrogen bonds with the corresponding *d*_norm_ contact distances being 1.62 and 1.64 Å, respectively, *i.e*. significantly shorter by almost 1 Å compared to the sum of the van der Waals radii of 2.61 Å (adjusted to neutron values), Table 2 ▸. Red spots of moderate intensity are observed for DMF-C15—H15⋯O2(carbon­yl) contact with a distance of 2.29 Å, while spots with weak to diminutive intensities are observed for other close contacts which mainly involve the aromatic rings and carb­oxy­lic groups of DTBA as well as the carbonyl group of DMF.

**Figure 5:**
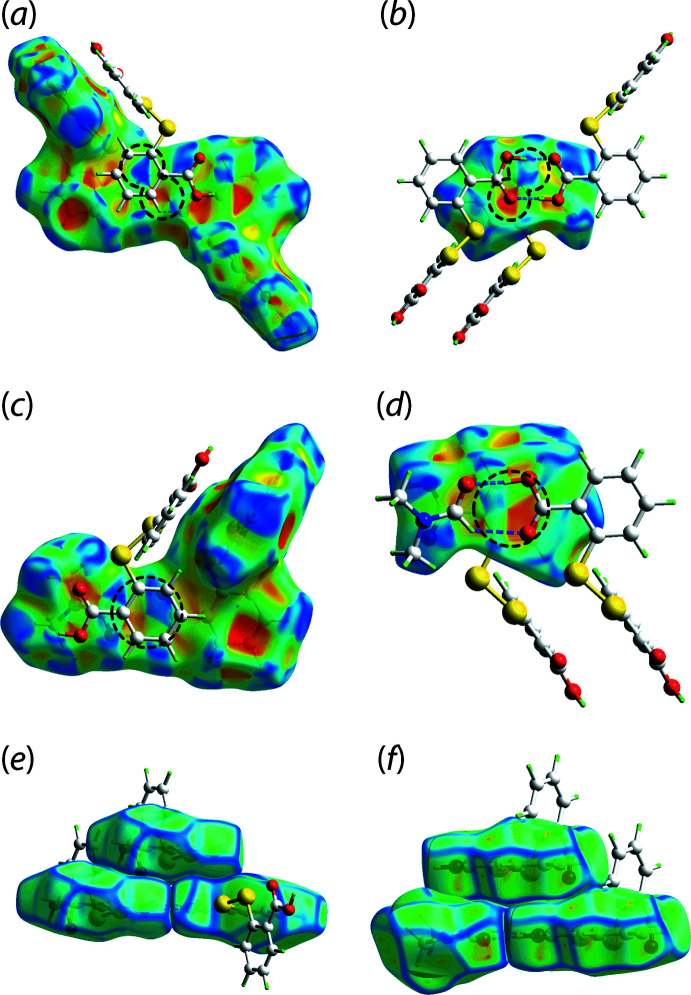
The Hirshfeld surface mapped with shape index (property range: −1.0 to +1.0 arbitrary units) for (*a*) a DTBA dimer, (*b*) a benzoic acid fragment in the opposite view of the DTBA dimer shown in (*a*), (*c*) a DTBA⋯DMF dimer and (*d*) a benzoic acid fragment in the opposite view of the DTBA⋯DMF dimer shown in (*c*). The Hirshfeld surface mapped with curvedness (property range: −4.0 to +0.4 arbitrary units) for the (*e*) π(C8–C13)⋯quasi-(⋯O4–C14–O3–H3*O*)_2_ inter­action and (*f*) π(C2–C7)⋯quasi-(O2–C1–O1–H1*O*⋯O5–C15–H15) inter­action. Both shape index and curvedness studies reveal the shape complementarity (as circled for the concave and convex represented by the red and blue regions in shape index) for the stacking arrangements between the corresponding ring systems.

Of particular inter­est among all close contacts present in (I) is a O3⋯C14 inter­action, which is included within an apparent *π*–*π* inter­action formed between the C8–C13 benzene ring and a quasi-*π*-system defined by O3—H3*O*⋯O4 hydrogen bonds between a DTBA dimer, *i.e*. the eight-membered {⋯O4–C14–O3–H3*O*}_2_ ring system. A similar observation is also noted for the C1⋯C15 contact which is encapsulated within an apparent *π*(C2–C7)⋯quasi-π(O2–C1–O1–H1*O*⋯O5–C15–H15) inter­action. The separation between the ring centroids of the aforementioned π–π contacts are 3.65 and 3.49 Å, respectively. The stacking arrangement between the relevant aromatic and quasi-aromatic rings is supported by shape complementarity as revealed by the concave (red) and convex (blue) regions in the shape index, Fig. 5 ▸(*a*)–(*d*), as well as curvedness mappings, Fig. 5 ▸(*e*) and (*f*), obtained through the Hirshfeld surface analysis.

The electrostatic potential property was mapped onto the Hirshfeld surface using the DFT-B3LYP/6-31G(*d*,*p*) approach to verify the nature of the contacts present in (I). The electrostatic charges for the points of contacts between each H-atom donor and acceptor are collated in Table 3 ▸. The results show that those inter­actions involving H-donors and O-acceptors are electrostatic in nature owing to the relatively great charge disparity between inter­acting atoms, with the greatest disparity being observed for the H1*O*⋯O5 followed by H3*O*⋯O4 inter­actions which is consistent with their corresponding short contact distances. By contrast, for the H⋯C and C⋯O inter­actions relatively smaller charge disparity is noted indicating weaker attractions between the participating atoms,. The exception is found for the C⋯C contacts which exhibit positive electrostatic charge for both donor and acceptor atoms signifying the dispersive nature of the contacts.

**Figure 6:**
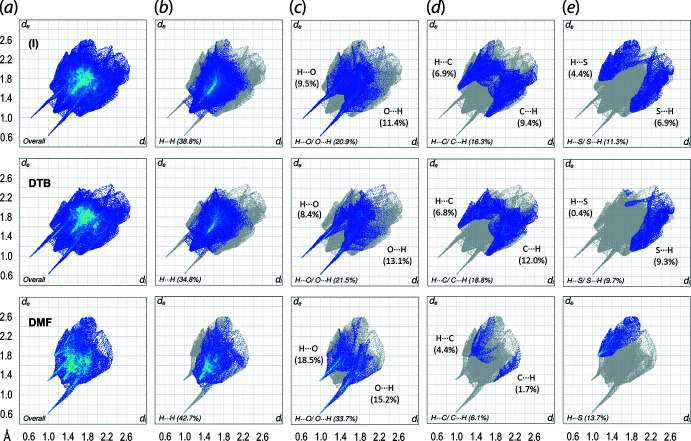
(*a*) The overall two-dimensional fingerprint plots for (I) (upper view), DTBA (middle) and DMF (lower) showing the corresponding overall fingerprint profiles as well as those delineated into (*b*) H⋯H, (*c*) H⋯O/ O⋯H, (*d*) H⋯C/ C⋯H and (*e*) H⋯S/ S⋯H contacts, with the percentage contributions being specified for each contact indicated therein.

The qu­anti­fication of the corresponding close contacts on the Hirshfeld surface through fingerprint plot analysis for overall (I) and its individual components, Fig. 6 ▸, show that the distributions mainly comprise H⋯H [(I): 38.8%; DTBA: 34.8%; DMF: 42.7%], H⋯O/O⋯H [(I): 20.9%; DTBA: 21.5%; DMF: 33.7%], H⋯C/C⋯H [(I): 16.3%; DTBA: 18.8%; DMF: 6.1%] and H⋯S/S⋯H [(I): 11.3%; DTBA: 9.7%; DMF: 13.7%]. The distinctive peaks of the minimum *d*_i_ + *d*_e_ values for H⋯O/O⋯H contacts correspond to O1—H1*O*⋯O5, O3—H3*O*⋯O4 and C15—H15⋯O2, and for the H⋯C/C⋯H contacts, to C5—H5⋯C11 and C11—H11⋯C6, while the peaks for H⋯S/ S⋯H exhibit a *d*_i_ + *d*_e_ contact distance of ∼2.92 Å, which is slightly shorter than the sum of the van der Waals radii (∑vdW radii) of 2.89 Å, Fig. 6 ▸(*e*). Further delineation of H⋯O/O⋯H, H⋯C/C⋯H and H⋯S/S⋯H shows that those heterogeneous contacts are more inclined towards (inter­nal)-*X*⋯H-(external) in DTBA, while the opposite is true for DMF indicating the complementary H-bond accepting and donating nature of DTBA and DMF, respectively. The inclination is more towards (inter­nal)-*X*⋯H-(external) for (I) which reflects the relatively small exposed surface for the DMF mol­ecule and limited hydrogen-bond donating role in the overall mol­ecular packing.

## Computational chemistry   

**Figure 7:**
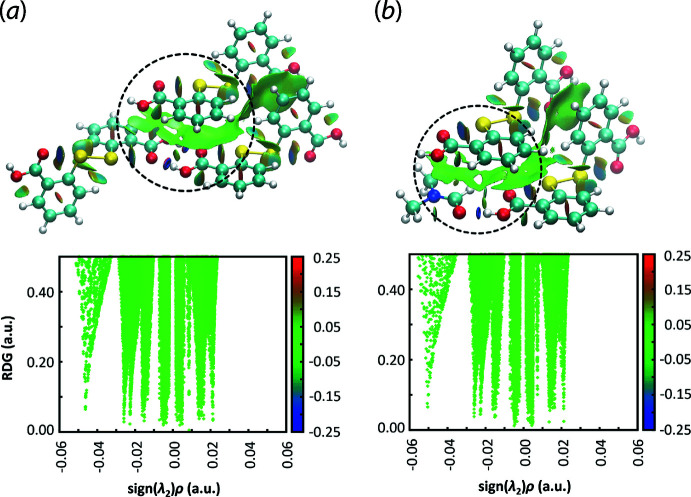
The non-covalent inter­action and corresponding RDG versus sign(λ^2^)ρ plots for the (*a*) π(C8–C13)⋯quasi-(⋯O4–C14–O3–H3*O*)_2_ inter­action and (*b*) π(C2–C7)⋯quasi-(O2–C1–O1–H1*O*⋯O5–C15–H15) inter­action. Both inter­actions are circled in black.

The program *NCIPLOT* (Johnson *et al.*, 2010 ▸) was employed to verify the non-covalent contacts for the π(C8–C13)–quasi-π(⋯O4–C14–O3–H3*O*)_2_ and π(C2–C7)–quasi-π(O2–C1–O1–H1*O*⋯O5–C15–H15) inter­actions as detected in the Hirshfeld surface analysis by calculating the electron density derivatives through wavefunction approach. The visualization of the resulting gradient isosurface supported the existence of the π–quasi-π contacts based on the corresponding large green domain sandwiched between the aromatic and quasi-aromatic rings. The overall density is in the range of −0.05 < sign(λ^2^)ρ < 0.03 a.u. indicating a weak but attractive inter­action (Contreras-García *et al.*, 2011 ▸), Fig. 7 ▸.

The strength of each close contact between all pairwise mol­ecules in (I) was qu­anti­fied through the calculation of the inter­action energies using *Crystal Explorer 17* (Turner *et al.*, 2017 ▸). As expected, the conventional hy­droxy-O3—H3*O*⋯O4(carbon­yl) hydrogen bond, leading to the eight-membered homosynthon as well as the seven-membered heterosynthon formed between hy­droxy-O1—H1*O*⋯O5(carbon­yl) and DMF-C15—H15⋯O2(carbon­yl) exhibit the greatest inter­action energies (*E*_int_) of −69.8 and −58.9 kJ mol^−1^, respectively. These are relatively stronger than the other supplementary contacts in (I), in which the corresponding energy terms, *viz*. electrostatic (*E*_ele_), polarization (*E*_pol_), dispersion (*E*_dis_), exchange-repulsion (*E*_rep_) together with the total energy are collated in Table 4 ▸.

Complementing the calculations with *Crystal Explorer* 17, the *E*_int_ for the pairs of π⋯quasi-π inter­actions were modelled in *Gaussian16* (Frisch *et al.*, 2016 ▸) by subjecting the respective three-mol­ecule aggregates as well as the hydrogen-bonded dimers, as shown in Fig. 7 ▸, for gas-phase energy calculation through a long-range corrected ωB97XD functional combining the D2 version of Grimme’s dispersion model (Chai & Head-Gordon, 2008 ▸) and coupled with Ahlrichs’s valence triple-zeta polarization basis sets (ωB97XD/def2-TZVP) (Weigend & Ahlrichs, 2005 ▸). Counterpoise methods (Boys & Bernardi, 1970 ▸; Simon *et al.*, 1996 ▸) were applied to correct for basis set superposition error (BSSE) in the obtained energies. The corresponding three-mol­ecule aggregates exhibit the greatest stabilization energy with the *E* being −132.5 and −119.7 kJ mol^−1^, respectively, which is consistent with the large localized green domains as detected through *NCIPLOT*. Upon the subtraction of the *E* contributed by the hydrogen bonded dimers*, i.e*. −73.2 kJ mol^−1^ for {⋯OCOH}_2_ and −60.5 kJ mol^−1^ for {⋯OCOH⋯OCH}, the remaining energies are ascribed to the π(C8–C13)⋯quasi-π(⋯O4–C14–O3–H3*O*)_2_ or π(C2–C7)⋯quasi-π(O2–C1–O1–H1*O*⋯O5–C15–H15) inter­actions, *i.e*. −59.3 and −59.2 kJ mol^−1^, respectively.

**Figure 8:**
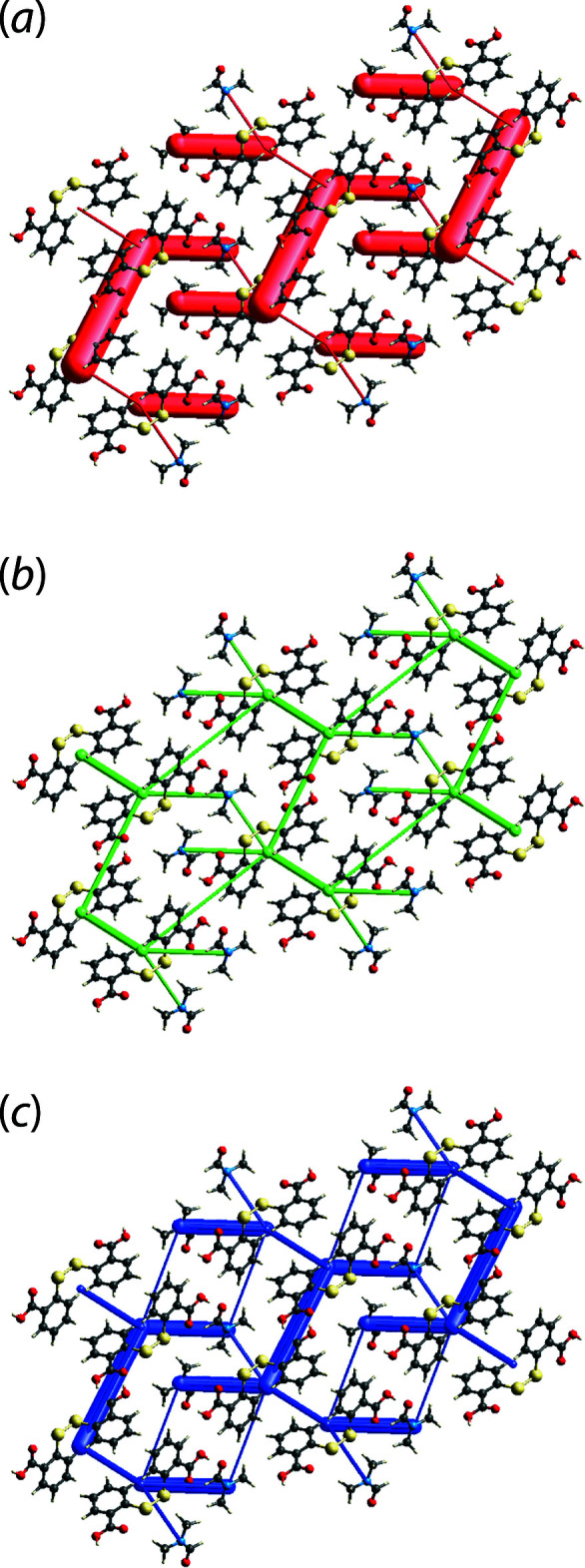
The energy frameworks for (I) viewed along the *a* axis, showing the (*a*) electrostatic force, (*b*) dispersion force and (*c*) total energy diagram. The cylindrical radius is proportional to the relative strength of the corresponding energies and they were adjusted to the same scale factor of 100 with a cut-off value of 8 kJ mol^−1^ within a 2 × 2 × 2 unit cells.

The crystal of (I) is predominantly governed by electrostatic force attributed to the strong O—H⋯O hydrogen-bonding contacts that lead to a maze-like *E*_ele_ topological framework as shown in Fig. 8 ▸(*a*). On the other hand, the dispersion force sustained by the specified π–π inter­actions results in a boat-shape topology, Fig. 8 ▸(*b*). The combination of the electrostatic and dispersion forces supersedes the strong inter­action energy from O—H⋯O contacts and lead to a refined overall energy framework with razor-blade-like topology, Fig. 8 ▸(*c*).

## Comparison of (I) with the di-DMF solvate   

The crystal structure of DTBA·2DMF (II) is also known, being reported four times (XEBDEO: Cai *et al.*, 2006 ▸; XEBDEO01: Ma *et al.*, 2013 ▸; AYIVAH: Baruah, 2016 ▸; CUNJUT: Tan & Tiekink, 2020 ▸). The key feature of the mol­ecular packing of (II) is that each carb­oxy­lic acid residue of the DTBA acid mol­ecule, which lacks crystallographic symmetry, is hydrogen bonded to a DMF mol­ecule to form a three-mol­ecule aggregate. For comparison purposes, (II) (CUNJUT: Tan & Tiekink, 2020 ▸), which was evaluated under similar experimental conditions as (I), was also subjected to mol­ecular packing and contact distribution studies. The calculation of the solvent accessible void space using the parameters as mentioned previously shows that the inclusion of additional DMF mol­ecules in the unit-cell is almost directly proportional to the occupied volume by the solvent mol­ecule, *i.e*. occupied unit-cell volume = 220.8 Å^3^ = 25.4% for (I) and 526.4 Å^3^ and 47.5% for (II).

**Figure 9:**
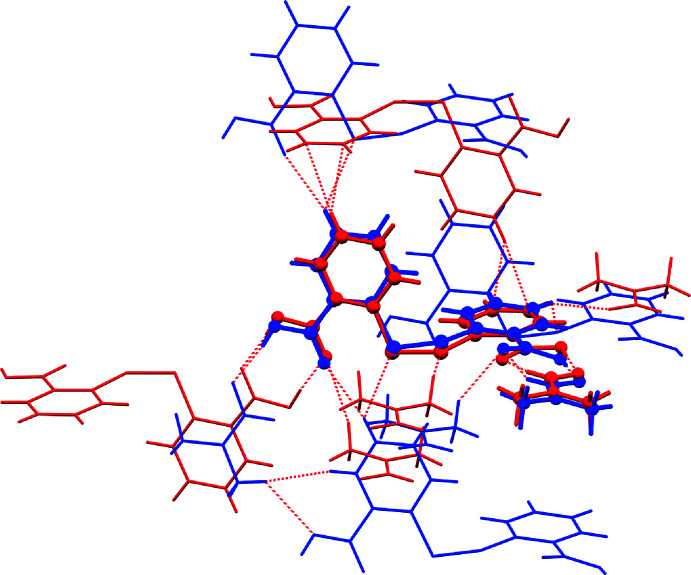
A comparison of crystal packing similarity within a 20% geometric tolerance between (I) (red trace) and (II) (blue) with the overlapped mol­ecules represented in ball-and-stick mode.

An analysis of the molecular packing similarity between (I) and (II) demonstrates that although the crystal solvates contain DTBA mol­ecule in common, the inclusion of additional DMF results results in a significant deviation in the mol­ecular packing as evidenced in Fig. 9 ▸. Here, only two out of 15 mol­ecules in the cluster of mol­ecules being studied are overlapped (within 20% geometric tolerance), with the r.m.s. deviation of the mol­ecular packing being 0.337 Å.

**Figure 10:**
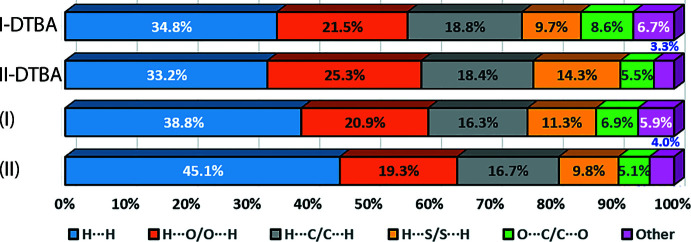
A comparison of the percentage contributions of various contacts to the Hirshfeld surfaces for (*a*) DTBA in (I), (*b*) DTBA in (II), (*c*) (I) and (*d*) (II).

In term of contact distribution on the Hirshfeld surface for the corresponding individual DTBA mol­ecules and overall (I) and (II), it is noted there are no great disparities in the percentage contributions to the calculated surfaces, Fig. 10 ▸.

## Database survey   

**Figure 11:**
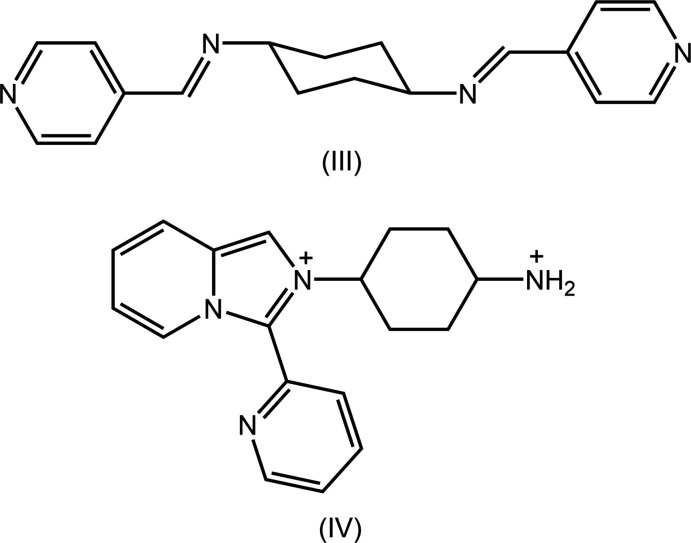
Chemical diagrams for (III) and (IV).

As mentioned in the *Chemical Context*, DTBA is usually generated during co-crystallization experiments with 2-mercapto­benzoic acid (2-MBA), implying oxidation of the latter. In addition to oxidation of 2-MBA, other crystallization outcomes have been observed during recent experiments suggesting chemical reactions are occurring. A less common outcome of crystallization experiments with 2-MBA was the sulfur extrusion product, 2,2′-thiodi­benzoic acid (Gorobet *et al.*, 2018 ▸), obtained during attempts to react 2-MBA with copper(I) chloride in the presence of two equivalents of tri­phenyl­phosphane (Tan & Tiekink, 2018 ▸). In a series of experiments with the isomeric Schiff bases, *N*,*N*-bis­[(pyridine-*n*-yl)methyl­ene]cyclo­hexane-1,4-di­amine, for *n* = 2, 3 and 4 (Lai *et al.*, 2006 ▸), very different products have been characterized from comparable reaction conditions. Referring to Fig. 11 ▸, (III) is the *n* = 4 isomer. Thus, when (III) was co-crystallized with 2-MBA, a salt of composition [1,4-H_3_N^(+)^C_6_H_10_N^(+)^H_3_][DTBA_2H]·DMF·H_2_O was isolated (KOZSOK; Tan & Tiekink, 2019*f* ▸). A more dramatic outcome was the cation, (IV), in the salt hydrate formulated as (IV)[DTBA_2H]·2H_2_O, where (IV) is 2-(4-ammonio­cyclo­hex­yl)-3-(pyridin-2-yl)imidazo[1,5-*a*]pyridin-2-ium di-cation, isolated from the co-crystallization of 2-MBA with the *n* = 2 isomer of (III) (TOLLEO; Tan & Tiekink, 2019*e* ▸). When 4-MBA was employed with the *n* = 2 isomer, [1,4-H_3_N^(+)^C_6_H_10_N^(+)^H_3_][4-DTBA_2H]·DMSO·H_2_O was the crystallization product (WOVHOH; Tan & Tiekink, 2019*g* ▸). Simple co-crystallization of 4-MBA with the 4-isomer gave the anti­cipated co-crystal [4-DTBA](II) (GOQREM; Tan & Tiekink, 2019*h* ▸). The aforementioned crystallization outcomes vindicate continued systematic investigations in this field.

## Synthesis and crystallization   

The DMF monosolvate of DTBA, (I), was obtained by the addition of a small amount of DMF to the benzene solution of 2-mercapto­benzoic acid (1 ml DMF: 5 ml benzene), followed by slow evaporation of the solvent. M.p. 462.5–463.7 K. IR (cm^−1^): 3072 *ν*(C—H), 1680 *ν*(C=O), 1464 *ν*(C=C), 1410 *δ*(C—H), 722 *ν*(C—S).

## Refinement   

Crystal data, data collection and structure refinement details are summarized in Table 5 ▸. The carbon-bound H atoms were placed in calculated positions (C—H = 0.95–0.98 Å) and were included in the refinement in the riding model approximation, with *U*_iso_(H) set to 1.2*U*_eq_(C). The oxygen-bound H atoms were located from a difference-Fourier map and refined with O—H = 0.84±0.01 Å, and with *U*_iso_(H) set to 1.5*U*_eq_(O).

## Supplementary Material

Crystal structure: contains datablock(s) I. DOI: 10.1107/S2056989020008257/hb7925sup1.cif

Structure factors: contains datablock(s) I. DOI: 10.1107/S2056989020008257/hb7925Isup2.hkl

Supporting information file. DOI: 10.1107/S2056989020008257/hb7925Isup3.cml

CCDC reference: 2011285

Additional supporting information:  crystallographic information; 3D view; checkCIF report

## Figures and Tables

**Table 1:** Hydrogen-bond geometry (Å, °) *Cg*1 and *Cg*2 are the centroids of the (C2–C7) and (C8–C13) rings, respectively.

*D*—H⋯*A*	*D*—H	H⋯*A*	*D*⋯*A*	*D*—H⋯*A*
O1—H1*O*⋯O5	0.85 (1)	1.75 (1)	2.5981 (13)	176 (2)
O3—H3*O*⋯O4^i^	0.84 (2)	1.78 (2)	2.6215 (13)	175 (2)
C15—H15⋯O2	0.95	2.38	3.1162 (15)	134
C7—H7⋯O1^ii^	0.95	2.53	3.2850 (16)	136
C1—O2⋯*Cg*1^iii^	1.22 (1)	3.42 (1)	3.4843 (12)	83 (1)
C14—O4⋯*Cg*2^iv^	1.23 (1)	3.33 (1)	3.6227 (12)	94 (1)
C11—H11⋯*Cg*1^v^	0.95	2.94	3.7962 (14)	150

Symmetry codes: (i) 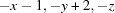; (ii) 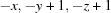; (iii) 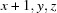; (iv) 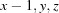; (v) 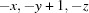.

**Table 2:** A summary *d*_norm_ contact distances (adjusted to neutron values) for inter­actions present in the crystal of (I) as computed through a Hirshfeld surface analysis

Contact	Distance	ΣvdW^*a*^	Δ|(*d*_norm_ − ΣvdW)|	Symmetry operation
H1*O*⋯O5^*b*^	1.62	2.61	0.99	*x*, *y*, *z*
H3*O*⋯O4^*b*^	1.64	2.61	0.97	−1 − *x*, 2 − *y*, −*z*
O2⋯H15	2.29	2.61	0.32	*x*, *y*, *z*
H7⋯O1	2.44	2.61	0.17	−*x*, 1 − *y*, 1 − *z*
H5⋯C11	2.64	2.79	0.15	−1 − *x*, 1 − *y*, − *z*
H11⋯C6	2.66	2.79	0.13	−*x*, 1 − *y*, −*z*
C1⋯C15	3.28	3.40	0.12	−1 + *x*, *y*, *z*
H6⋯O5	2.49	2.61	0.12	−*x*, 1 − *y*, 1 − *z*
H11⋯C5	2.68	2.79	0.11	−*x*, 1 − *y*, −*z*
O4⋯H16*A*	2.53	2.61	0.08	1 − *x*, 2 − *y*, 1 − *z*
O3⋯C14	3.17	3.22	0.05	−*x*, 2 − *y*, −*z*
C14⋯C14	3.37	3.40	0.03	−*x*, 2 − *y*, −*z*

Notes: (*a*) ΣvdW is the sum of the respective van der Waals radii; (*b*) these inter­actions correspond to conventional hydrogen bonds.

**Table 3:** Electrostatic potential charge (*V*_ESP_) for each hydrogen-atom donor and acceptor in (I) participating in a close contact identified through the Hirshfeld surface analysis

Contact	Electrostatic potential, *V*_ESP_ (a.u.)		Δ|*V*_ESP_|
	H-donor	H-acceptor	
H1*O*⋯O5	0.2757	−0.0854	0.3611
H3*O*⋯O4	0.2622	−0.0476	0.3098
H6⋯O5	0.0394	−0.0875	0.1269
H16*A*⋯O4	0.0366	−0.0669	0.1035
H15⋯O2	0.0362	−0.0605	0.0967
H7⋯O1	0.0373	−0.0249	0.0622
H11⋯C6	0.0465	−0.0080	0.0545
H11⋯C5	0.0431	−0.0068	0.0499
H5⋯C11	0.0446	−0.0016	0.0462
C14⋯O3	0.0192	−0.0080	0.0272
C1⋯C15	0.0238	0.0161	0.0077
C14⋯C14	0.0196	0.0191	0.0005

**Table 4:** A summary of inter­action energies (kJ mol^−1^) calculated for (I)

Contact	*E*_ele_	*E*_pol_	*E*_dis_	*E*_rep_	*E*_tot_	symmetry operation
{O3—H3*O*⋯O4}_2_	−135.2	−21.5	−12.1	99.1	−69.8	−1 − *x*, 2 − *y*, − *z*
O1—H1*O*⋯O5 +						
C15—H15⋯O2	−94.8	−15.8	−9.5	61.3	−58.9	*x*, *y*, *z*
{C11—H11⋯π(C2–C7)}_2_	−10.6	−0.8	−30.5	17.7	−24.2	−*x*, 1 − *y*, −*z*
{C14⋯O3}_2_ +						
C14⋯C14	−7.0	−1.2	−20.3	7.1	−21.5	−*x*, 2 − *y*, −*z*
C1⋯C15	−6.4	−2.1	−18.5	7.0	−19.9	−1 + *x*, *y*, *z*
C16—H16*A*⋯O4	−9.9	−1.6	−12.5	9.5	−14.6	1 − *x*, 2 − *y*, 1 − *z*
{C5—H5⋯π(C8–C13)}_2_	−6.0	−0.6	−22.6	12.1	−14.2	−1 − *x*, 1 − *y*, −*z*
C6—H6⋯O5	−7.0	−2.0	−19.7	3.0	−9.5	−*x*, 1 − *y*, 1 − *z*
C7—H7⋯O1	−3.8	−0.8	−12.6	10.1	−7.2	−*x*, 1 − *y*, 1 − *z*

**Table 5:** Experimental details

Crystal data	
Chemical formula	C_14_H_10_O_4_S_2_·C_3_H_7_NO
*M*_r_	379.43
Crystal system, space group	Triclinic, *P* 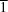
Temperature (K)	100
*a*, *b*, *c* (Å)	5.05866 (4), 12.2617 (1), 15.1009 (1)
α, β, γ (°)	106.149 (1), 96.446 (1), 100.884 (1)
*V* (Å^3^)	869.94 (1)
*Z*	2
Radiation type	Cu *K*α
μ (mm^−1^)	3.03
Crystal size (mm)	0.24 × 0.16 × 0.06

Data collection	
Diffractometer	XtaLAB Synergy, Dualflex, AtlasS2
Absorption correction	Gaussian (*CrysAlis PRO*; Rigaku OD, 2018 ▸)
*T*_min_, *T*_max_	0.316, 1.000
No. of measured, independent and observed [*I* > 2σ(*I*)] reflections	19670, 3543, 3410
*R*_int_	0.025
(sin θ/λ)_max_ (Å^−1^)	0.630

Refinement	
*R*[*F* ^2^ > 2σ(*F* ^2^)], *wR*(*F* ^2^), *S*	0.026, 0.072, 1.07
No. of reflections	3543
No. of parameters	234
No. of restraints	2
H-atom treatment	H atoms treated by a mixture of independent and constrained refinement
Δρ_max_, Δρ_min_ (e Å^−3^)	0.23, −0.34

Computer programs: *CrysAlis PRO* (Rigaku OD, 2018 ▸), *SHELXS* (Sheldrick, 2015*a* ▸), *SHELXL2017/1* (Sheldrick, 2015*b* ▸), *ORTEP-3 for Windows* (Farrugia, 2012 ▸), *DIAMOND* (Brandenburg, 2006 ▸) and *publCIF* (Westrip, 2010 ▸).

## supplementary crystallographic information

### Crystal data

**Table:**

C_14_H_10_O_4_S_2_·C_3_H_7_NO	*Z* = 2
*M**_r_* = 379.43	*F*(000) = 396
Triclinic, *P*1	*D*_x_ = 1.449 Mg m^−^^3^
*a* = 5.05866 (4) Å	Cu *K*α radiation, λ = 1.54184 Å
*b* = 12.2617 (1) Å	Cell parameters from 13143 reflections
*c* = 15.1009 (1) Å	θ = 3.1–76.0°
α = 106.149 (1)°	µ = 3.03 mm^−^^1^
β = 96.446 (1)°	*T* = 100 K
γ = 100.884 (1)°	Prism, colourless
*V* = 869.94 (1) Å^3^	0.24 × 0.16 × 0.06 mm

### Data collection

**Table:**

XtaLAB Synergy, Dualflex, AtlasS2 diffractometer	3410 reflections with *I* > 2σ(*I*)
Detector resolution: 5.2558 pixels mm^-1^	*R*_int_ = 0.025
ω scans	θ_max_ = 76.3°, θ_min_ = 3.1°
Absorption correction: gaussian (CrysAlisPro; Rigaku OD, 2018)	*h* = −6→6
*T*_min_ = 0.316, *T*_max_ = 1.000	*k* = −15→13
19670 measured reflections	*l* = −18→18
3543 independent reflections	

### Refinement

**Table:**

Refinement on *F*^2^	Primary atom site location: dual
Least-squares matrix: full	Hydrogen site location: mixed
*R*[*F*^2^ > 2σ(*F*^2^)] = 0.026	H atoms treated by a mixture of independent and constrained refinement
*wR*(*F*^2^) = 0.072	*w* = 1/[σ^2^(*F*_o_^2^) + (0.0405*P*)^2^ + 0.3204*P*] where *P* = (*F*_o_^2^ + 2*F*_c_^2^)/3
*S* = 1.07	(Δ/σ)_max_ = 0.001
3543 reflections	Δρ_max_ = 0.23 e Å^−^^3^
234 parameters	Δρ_min_ = −0.34 e Å^−^^3^
2 restraints	

### Special details

**Table:**

Geometry. All esds (except the esd in the dihedral angle between two l.s. planes) are estimated using the full covariance matrix. The cell esds are taken into account individually in the estimation of esds in distances, angles and torsion angles; correlations between esds in cell parameters are only used when they are defined by crystal symmetry. An approximate (isotropic) treatment of cell esds is used for estimating esds involving l.s. planes.

### Fractional atomic coordinates and isotropic or equivalent isotropic displacement parameters (Å^2^)

**Table:**

	*x*	*y*	*z*	*U*_iso_*/*U*_eq_	
S1	0.13127 (6)	0.80270 (2)	0.28401 (2)	0.01841 (9)	
S2	−0.10332 (6)	0.84945 (2)	0.18710 (2)	0.01848 (9)	
O1	0.32810 (18)	0.61831 (8)	0.48410 (6)	0.02255 (19)	
H1O	0.480 (2)	0.6544 (14)	0.5192 (11)	0.034*	
O2	0.42509 (17)	0.76207 (8)	0.41991 (6)	0.02129 (19)	
O3	−0.30085 (18)	0.92323 (8)	−0.07873 (6)	0.02025 (19)	
H3O	−0.403 (3)	0.9709 (12)	−0.0714 (12)	0.030*	
O4	−0.37132 (17)	0.93328 (7)	0.06697 (6)	0.01850 (18)	
O5	0.78590 (18)	0.72556 (8)	0.59804 (6)	0.0248 (2)	
N1	1.1659 (2)	0.87050 (9)	0.62312 (7)	0.0212 (2)	
C1	0.2742 (2)	0.67339 (10)	0.42316 (8)	0.0176 (2)	
C2	0.0090 (2)	0.61916 (10)	0.35774 (8)	0.0170 (2)	
C3	−0.0765 (2)	0.67004 (10)	0.28992 (8)	0.0166 (2)	
C4	−0.3247 (2)	0.61624 (11)	0.22897 (9)	0.0193 (2)	
H4	−0.380732	0.647747	0.181123	0.023*	
C5	−0.4903 (2)	0.51701 (11)	0.23773 (9)	0.0213 (3)	
H5	−0.660717	0.482386	0.196749	0.026*	
C6	−0.4093 (3)	0.46787 (11)	0.30573 (9)	0.0219 (3)	
H6	−0.524200	0.400478	0.311867	0.026*	
C7	−0.1591 (3)	0.51829 (11)	0.36451 (8)	0.0204 (2)	
H7	−0.100899	0.483843	0.410105	0.024*	
C8	−0.0191 (2)	0.77686 (10)	0.07734 (8)	0.0165 (2)	
C9	−0.0966 (2)	0.80827 (10)	−0.00313 (8)	0.0156 (2)	
C10	−0.0139 (2)	0.75656 (11)	−0.08709 (9)	0.0192 (2)	
H10	−0.062982	0.779179	−0.140805	0.023*	
C11	0.1383 (3)	0.67300 (11)	−0.09306 (9)	0.0222 (3)	
H11	0.196005	0.639201	−0.150100	0.027*	
C12	0.2057 (3)	0.63912 (11)	−0.01461 (9)	0.0225 (3)	
H12	0.306044	0.580290	−0.018626	0.027*	
C13	0.1282 (2)	0.69023 (11)	0.06944 (9)	0.0200 (2)	
H13	0.175946	0.665950	0.122388	0.024*	
C14	−0.2676 (2)	0.89386 (10)	−0.00130 (8)	0.0152 (2)	
C15	0.9281 (2)	0.80364 (11)	0.57469 (9)	0.0207 (2)	
H15	0.861275	0.816264	0.517895	0.025*	
C16	1.2863 (3)	0.85536 (13)	0.70996 (9)	0.0282 (3)	
H16A	1.268974	0.919308	0.763221	0.042*	
H16B	1.480062	0.855771	0.709563	0.042*	
H16C	1.190793	0.780820	0.715480	0.042*	
C17	1.3127 (3)	0.96362 (12)	0.59254 (10)	0.0281 (3)	
H17A	1.220139	0.959343	0.530664	0.042*	
H17B	1.500247	0.954934	0.588962	0.042*	
H17C	1.316721	1.039287	0.637404	0.042*	

### Atomic displacement parameters (Å^2^)

**Table:**

	*U*^11^	*U*^22^	*U*^33^	*U*^12^	*U*^13^	*U*^23^
S1	0.01995 (15)	0.01712 (15)	0.01807 (15)	0.00257 (11)	−0.00087 (11)	0.00800 (11)
S2	0.02349 (16)	0.01848 (15)	0.01612 (15)	0.00924 (11)	0.00252 (11)	0.00694 (11)
O1	0.0220 (4)	0.0274 (5)	0.0201 (4)	0.0019 (4)	−0.0003 (3)	0.0142 (4)
O2	0.0223 (4)	0.0213 (4)	0.0196 (4)	0.0009 (3)	−0.0004 (3)	0.0097 (3)
O3	0.0250 (4)	0.0239 (5)	0.0179 (4)	0.0124 (4)	0.0059 (3)	0.0106 (3)
O4	0.0215 (4)	0.0206 (4)	0.0170 (4)	0.0096 (3)	0.0043 (3)	0.0078 (3)
O5	0.0235 (4)	0.0279 (5)	0.0236 (5)	0.0015 (4)	0.0021 (4)	0.0127 (4)
N1	0.0222 (5)	0.0218 (5)	0.0182 (5)	0.0039 (4)	0.0032 (4)	0.0049 (4)
C1	0.0207 (6)	0.0198 (6)	0.0141 (5)	0.0069 (5)	0.0049 (4)	0.0061 (4)
C2	0.0181 (6)	0.0195 (6)	0.0143 (5)	0.0054 (4)	0.0049 (4)	0.0050 (4)
C3	0.0166 (5)	0.0167 (5)	0.0175 (6)	0.0056 (4)	0.0051 (4)	0.0049 (4)
C4	0.0181 (6)	0.0206 (6)	0.0195 (6)	0.0071 (5)	0.0025 (5)	0.0054 (5)
C5	0.0167 (5)	0.0216 (6)	0.0224 (6)	0.0036 (5)	0.0029 (5)	0.0024 (5)
C6	0.0224 (6)	0.0194 (6)	0.0227 (6)	0.0012 (5)	0.0082 (5)	0.0052 (5)
C7	0.0250 (6)	0.0208 (6)	0.0173 (6)	0.0053 (5)	0.0066 (5)	0.0078 (5)
C8	0.0148 (5)	0.0156 (5)	0.0184 (6)	0.0026 (4)	0.0018 (4)	0.0052 (4)
C9	0.0130 (5)	0.0142 (5)	0.0187 (6)	0.0018 (4)	0.0013 (4)	0.0053 (4)
C10	0.0184 (6)	0.0195 (6)	0.0191 (6)	0.0034 (4)	0.0026 (4)	0.0057 (5)
C11	0.0214 (6)	0.0214 (6)	0.0225 (6)	0.0067 (5)	0.0061 (5)	0.0026 (5)
C12	0.0195 (6)	0.0184 (6)	0.0297 (7)	0.0082 (5)	0.0038 (5)	0.0052 (5)
C13	0.0188 (6)	0.0187 (6)	0.0235 (6)	0.0056 (5)	0.0013 (5)	0.0083 (5)
C14	0.0146 (5)	0.0142 (5)	0.0156 (5)	0.0010 (4)	0.0002 (4)	0.0053 (4)
C15	0.0215 (6)	0.0235 (6)	0.0174 (6)	0.0059 (5)	0.0031 (5)	0.0062 (5)
C16	0.0279 (7)	0.0346 (7)	0.0199 (6)	0.0089 (6)	−0.0014 (5)	0.0058 (5)
C17	0.0282 (7)	0.0219 (6)	0.0312 (7)	0.0004 (5)	0.0071 (6)	0.0060 (5)

### Geometric parameters (Å, º)

**Table:**

S1—C3	1.7929 (12)	C6—C7	1.3853 (18)
S1—S2	2.0524 (4)	C6—H6	0.9500
S2—C8	1.7894 (12)	C7—H7	0.9500
O1—C1	1.3177 (15)	C8—C13	1.3951 (16)
O1—H1O	0.845 (9)	C8—C9	1.4103 (16)
O2—C1	1.2216 (15)	C9—C10	1.3988 (16)
O3—C14	1.3184 (14)	C9—C14	1.4772 (15)
O3—H3O	0.845 (9)	C10—C11	1.3831 (17)
O4—C14	1.2295 (14)	C10—H10	0.9500
O5—C15	1.2423 (16)	C11—C12	1.3887 (18)
N1—C15	1.3228 (17)	C11—H11	0.9500
N1—C17	1.4557 (17)	C12—C13	1.3855 (18)
N1—C16	1.4573 (17)	C12—H12	0.9500
C1—C2	1.4893 (16)	C13—H13	0.9500
C2—C7	1.3985 (17)	C15—H15	0.9500
C2—C3	1.4082 (17)	C16—H16A	0.9800
C3—C4	1.3958 (17)	C16—H16B	0.9800
C4—C5	1.3889 (18)	C16—H16C	0.9800
C4—H4	0.9500	C17—H17A	0.9800
C5—C6	1.3885 (18)	C17—H17B	0.9800
C5—H5	0.9500	C17—H17C	0.9800
			
C3—S1—S2	104.21 (4)	C10—C9—C14	118.89 (10)
C8—S2—S1	104.44 (4)	C8—C9—C14	121.39 (10)
C1—O1—H1O	109.1 (12)	C11—C10—C9	120.98 (11)
C14—O3—H3O	107.5 (11)	C11—C10—H10	119.5
C15—N1—C17	121.01 (11)	C9—C10—H10	119.5
C15—N1—C16	121.21 (11)	C10—C11—C12	119.16 (11)
C17—N1—C16	117.77 (11)	C10—C11—H11	120.4
O2—C1—O1	123.78 (11)	C12—C11—H11	120.4
O2—C1—C2	121.86 (11)	C11—C12—C13	120.69 (11)
O1—C1—C2	114.35 (10)	C11—C12—H12	119.7
C7—C2—C3	119.58 (11)	C13—C12—H12	119.7
C7—C2—C1	120.03 (11)	C12—C13—C8	120.87 (12)
C3—C2—C1	120.37 (11)	C12—C13—H13	119.6
C4—C3—C2	118.92 (11)	C8—C13—H13	119.6
C4—C3—S1	121.24 (9)	O4—C14—O3	123.20 (10)
C2—C3—S1	119.84 (9)	O4—C14—C9	122.27 (10)
C5—C4—C3	120.53 (11)	O3—C14—C9	114.53 (10)
C5—C4—H4	119.7	O5—C15—N1	124.80 (12)
C3—C4—H4	119.7	O5—C15—H15	117.6
C4—C5—C6	120.71 (11)	N1—C15—H15	117.6
C4—C5—H5	119.6	N1—C16—H16A	109.5
C6—C5—H5	119.6	N1—C16—H16B	109.5
C7—C6—C5	119.22 (11)	H16A—C16—H16B	109.5
C7—C6—H6	120.4	N1—C16—H16C	109.5
C5—C6—H6	120.4	H16A—C16—H16C	109.5
C6—C7—C2	120.98 (12)	H16B—C16—H16C	109.5
C6—C7—H7	119.5	N1—C17—H17A	109.5
C2—C7—H7	119.5	N1—C17—H17B	109.5
C13—C8—C9	118.50 (11)	H17A—C17—H17B	109.5
C13—C8—S2	121.28 (9)	N1—C17—H17C	109.5
C9—C8—S2	120.20 (9)	H17A—C17—H17C	109.5
C10—C9—C8	119.71 (11)	H17B—C17—H17C	109.5
			
O2—C1—C2—C7	−179.30 (11)	S1—S2—C8—C9	−166.35 (8)
O1—C1—C2—C7	0.13 (16)	C13—C8—C9—C10	−3.03 (17)
O2—C1—C2—C3	−0.45 (17)	S2—C8—C9—C10	175.70 (9)
O1—C1—C2—C3	178.99 (10)	C13—C8—C9—C14	176.08 (10)
C7—C2—C3—C4	−1.89 (17)	S2—C8—C9—C14	−5.18 (15)
C1—C2—C3—C4	179.25 (10)	C8—C9—C10—C11	1.39 (18)
C7—C2—C3—S1	177.41 (9)	C14—C9—C10—C11	−177.74 (11)
C1—C2—C3—S1	−1.45 (15)	C9—C10—C11—C12	0.97 (18)
S2—S1—C3—C4	6.15 (10)	C10—C11—C12—C13	−1.66 (19)
S2—S1—C3—C2	−173.13 (8)	C11—C12—C13—C8	−0.04 (19)
C2—C3—C4—C5	2.79 (17)	C9—C8—C13—C12	2.38 (18)
S1—C3—C4—C5	−176.49 (9)	S2—C8—C13—C12	−176.34 (9)
C3—C4—C5—C6	−1.54 (18)	C10—C9—C14—O4	172.13 (11)
C4—C5—C6—C7	−0.67 (18)	C8—C9—C14—O4	−6.99 (17)
C5—C6—C7—C2	1.57 (18)	C10—C9—C14—O3	−7.31 (15)
C3—C2—C7—C6	−0.29 (18)	C8—C9—C14—O3	173.56 (10)
C1—C2—C7—C6	178.58 (11)	C17—N1—C15—O5	−177.13 (12)
S1—S2—C8—C13	12.35 (11)	C16—N1—C15—O5	1.3 (2)

### Hydrogen-bond geometry (Å, º)

*Cg*1 and *Cg*2 are the centroids of the (C2–C7) and (C8–C13) rings, respectively.

**Table:**

*D*—H···*A*	*D*—H	H···*A*	*D*···*A*	*D*—H···*A*
O1—H1*O*···O5	0.85 (1)	1.75 (1)	2.5981 (13)	176 (2)
O3—H3*O*···O4^i^	0.84 (2)	1.78 (2)	2.6215 (13)	175 (2)
C15—H15···O2	0.95	2.38	3.1162 (15)	134
C7—H7···O1^ii^	0.95	2.53	3.2850 (16)	136
C1—O2···*Cg*1^iii^	1.22 (1)	3.42 (1)	3.4843 (12)	83 (1)
C14—O4···*Cg*2^iv^	1.23 (1)	3.33 (1)	3.6227 (12)	94 (1)
C11—H11···*Cg*1^v^	0.95	2.94	3.7962 (14)	150

Symmetry codes: (i) −*x*−1, −*y*+2, −*z*; (ii) −*x*, −*y*+1, −*z*+1; (iii) *x*+1, *y*, *z*; (iv) *x*−1, *y*, *z*; (v) −*x*, −*y*+1, −*z*.
